# Processivity vs. Beating: Comparing Cytoplasmic and Axonemal Dynein Microtubule Binding Domain Association with Microtubule

**DOI:** 10.3390/ijms20051090

**Published:** 2019-03-03

**Authors:** Nayere Tajielyato, Emil Alexov

**Affiliations:** Department of Physics and Astronomy, Clemson University, Clemson, SC 29630, USA; ntajiel@g.clemson.edu

**Keywords:** electrostatics, dynein, microtubule, binding, conformational changes and intrinsic disordered protein

## Abstract

This study compares the role of electrostatics in the binding process between microtubules and two dynein microtubule-binding domains (MTBDs): cytoplasmic and axonemal. These two dyneins are distinctively different in terms of their functionalities: cytoplasmic dynein is processive, while axonemal dynein is involved in beating. In both cases, the binding requires frequent association/disassociation between the microtubule and MTBD, and involves highly negatively charged microtubules, including non-structured C-terminal domains (E-hooks), and an MTBD interface that is positively charged. This indicates that electrostatics play an important role in the association process. Here, we show that the cytoplasmic MTBD binds electrostatically tighter to microtubules than to the axonemal MTBD, but the axonemal MTBD experiences interactions with microtubule E-hooks at longer distances compared with the cytoplasmic MTBD. This allows the axonemal MTBD to be weakly bound to the microtubule, while at the same time acting onto the microtubule via the flexible E-hooks, even at MTBD–microtubule distances of 45 Å. In part, this is due to the charge distribution of MTBDs: in the cytoplasmic MTBD, the positive charges are concentrated at the binding interface with the microtubule, while in the axonemal MTBD, they are more distributed over the entire structure, allowing E-hooks to interact at longer distances. The dissimilarities of electrostatics in the cases of axonemal and cytoplasmic MTBDs were found not to result in a difference in conformational dynamics on MTBDs, while causing differences in the conformational states of E-hooks. The E-hooks’ conformations in the presence of the axonemal MTBD were less restricted than in the presence of the cytoplasmic MTBD. In parallel with the differences, the common effect was found that the structural fluctuations of MTBDs decrease as either the number of contacts with E-hooks increases or the distance to the microtubule decreases.

## 1. Introduction

Cytoplasmic dynein is one of the three different families of motor proteins moving toward the minus end of cytoskeleton filaments (microtubules) to do diverse activities, such as transport cargos including proteins, organelles, and mRNAs in eukaryotic cells [1,2,3,4]. Axonemal dynein, on the other hand, is non-processive, and it causes the sliding of microtubules [5,6]. Although cytoplasmic and axonemal dynein share some structural similarities [7], their functions within the cell are different [8,9,10]. Cytoplasmic dyneins are composed of two identical chains, stepping processively along microtubules. In contrast, in cilia and flagella, the dyneins are involved in the beating of cilia and flagella. In both cases, the dyneins interact with microtubules via a microtubule-binding domain (MTBD), which adopts different conformations due to the signal provided by the linker and ATP (Adenosine triphosphate) -binding domain [11,12]. It has been shown that a change in registry (α and β) between two antiparallel coiled coil helices, called the stalk [13], alters the microtubule binding affinity of cytoplasmic MTBD [11,14]; however, this effect is small for axonemal MTBD [7].

Microtubules are made of α-tubulins and β-tubulins. While the structures of these tubulins are experimentally known, each tubulin has an intrinsically disordered region rich in Glu and Asp amino acids located on the C-terminal. These regions are called “E-hooks”, and their structures are not available. While unstructured and only a small part of the entire tubulin sequence, E-hooks are implicated in various biological mechanisms. Thus, it was demonstrated that E-hooks affect microtubule dynamics [15,16], including ending binding protein recognition of microtubule plus ends [17], the spastin severing of microtubules [18], and microtubule motor protein motility [19,20]. Furthermore, E-hooks were shown to regulate force production [21], kinetochore attachment, and diffusion along the mitotic spindle [22,23], and tau–microtubule interactions [24]. The E-hooks impact cytoplasmic dynein’s processivity and speed [19,25,26], and E-hooks affect the beat of flagellar motility [27]. In terms of binding, our previous work [28] demonstrated that the electrostatic force between E-hooks and the cytoplasmic MTBD provides a “guided soft-binding” for MTBD as it approaches the microtubule. All these facts indicate the importance of E-hooks for MTBD–microtubule interactions.

Computational investigations on dynein mechanochemistry have been reported before, with the primary goal of providing a model for the separation and reattachment of the MTBD to the microtubule [29]. Furthermore, a mechanism was proposed to explain how flagellum can bend in three dimensions [30], and the relevance to its processive and oscillatory sliding [31,32]. However, to the best of our knowledge, little attention has been paid to simulating the role of E-hooks. 

The primary focus of this study is to reveal the similarities and differences between E-hooks’ interactions with cytoplasmic and axonemal MTBDs, and consequentially the effect on the conformational states of both MTBDs and E-hooks. Since the microtubule is highly charged, special attention is paid to the role of electrostatics in MTBD–microtubule association. 

## 2. Results

The goal of this work is to compare the binding process of axonemal and cytoplasmic MTBDs to the microtubule and the role of E-hooks. This is done by comparing: (a) the electrostatic features of MTBDs binding to the microtubule in the presence of E-hooks, (b) the number of contacts between the corresponding dynein MTBD and E-hooks at various MTBD–microtubule distances, (c) the conformational changes of the MTBDs due to their interaction with the microtubule as the MTBD’s distance increases, (d) how the conformational states of MTBDs in an isolated state are related to those of MTBDs in a bound state and at particular distances, (e) the population of clusters of E-hooks at various distances, and (f) the binding energies of MTBDs and microtubules in the presence and absence of E-hooks.

### 2.1. Electrostatic Features of Axonemal and Cytoplasmic MTBDs 

**Figure 1:**
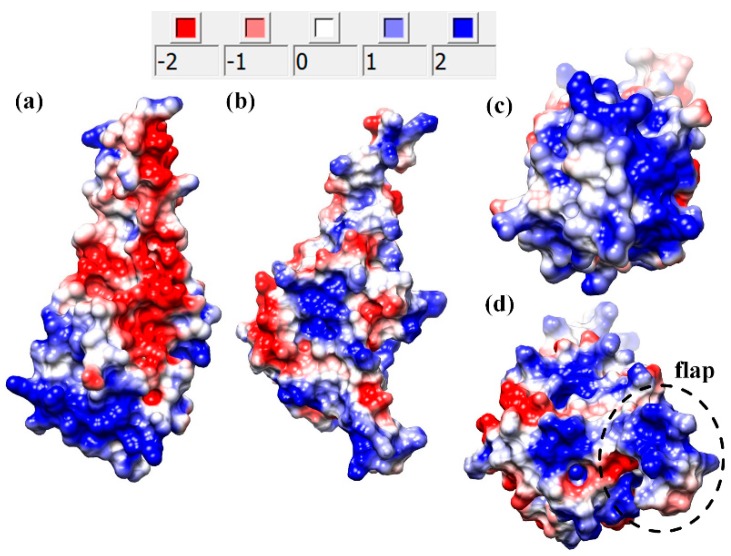
Electrostatic potential mapped onto the surface of microtubule-binding domains (MTBDs). Panels (**a**,**b**) show a side view of the electrostatic potential mapped onto the molecular surface of cytoplasmic and axonemal MTBDs, respectively. Panels (**c**,**d**) present the electrostatic potential on the binding interface for cytoplasmic and axonemal MTBDs, respectively. The flap region is indicated with a dashed circle. (The potential isocontour values, in term of kT/e (electrostatic potential unit), are shown in the legend).

The sequence of axonemal MTBD is 25% identical to the sequence of the cytoplasmic MTBD, and the axonemal MTBD is structurally similar to the cytoplasmic MTBD in a weak binding state (β-registry) (Figure S1a,b) [7]. Such high sequence and structural similarities suggest that axonemal and cytoplasmic MTBDs may have similar electrostatic features. To compare the electrostatic features of axonemal and cytoplasmic MTBDs, we first calculated the pKas of titratable residues using DelPhiPKa [33]. At pH = 7.0, all titratable residues, except His, were found to be fully ionized [33]. All glutamic acid and aspartic acid residues within the E-hooks were fully ionized at pH 7.0 and were kept charged in modeling protocol. Further, we found that the axonemal and cytoplasmic MTBDs have a net charge of +3 and 0 at pH = 7, respectively. At the same time, the microtubule itself is highly negatively charged, and the presence of E-hooks makes the charge even more negative. Since axonemal MTBD carries a positive net charge (net charge +3e) while cytoplasmic MTBD is neutral (net charge 0e) (a side view of electrostatic potential mapped onto the MTBD surface is shown in Figure 1a,b), it can be expected that the axonemal MTBD will interact stronger with the microtubule than the cytoplasmic one. However, the same polarity charges are much more clustered in cytoplasmic than in axonemal MTBD. Indeed, Figure 1a,b shows that positively and negatively charged patches are almost equally scattered over the surface of axonemal MTBD, while in the cytoplasmic MTBD, most of the positively charged patches are at the binding interface (Figure 1c). Thus, the binding interface of axonemal MTBD is less positively charged (Figure 1d) than that of cytoplasmic MTBD, with the exception of the flap region. Thus, without the flap region, the electrostatics are expected to be more favorable for cytoplasmic MTBD interactions with microtubules than for axonemal. 

**Figure 2:**
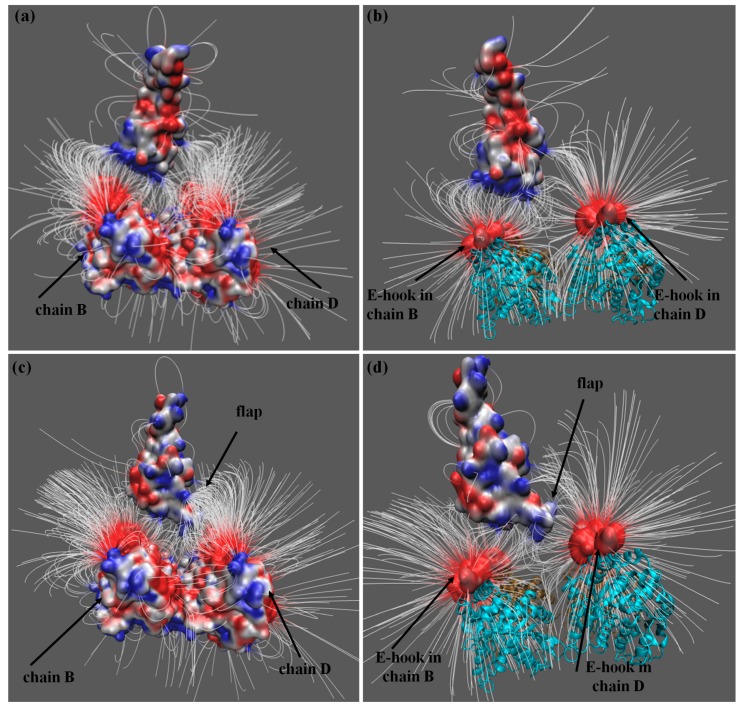
Electrostatic potential mapped onto the MTBD surface along with the electrostatic field lines around and between the corresponding MTBD and tubulins calculated with the corresponding MTBD separated from tubulins by 35 Å. Electrostatic field lines between MTBDs and all the residues of tubulins, including E-hooks, are shown in panel (**a**) for cytoplasmic dynein and (**c**) for axonemal dynein. Panels (**b**,**d**) present the electrostatic field lines between only E-hooks and the MTBDs of cytoplasmic and axonemal dyneins, respectively. The red and blue colors show the negative and positive electrostatic fields, respectively. Tubulins are shown with cyan ribbons.

While electrostatic potential at the binding interface provides insights about the role of electrostatic in the binding, further information can be obtained via the electrostatic field (which implies the electrostatic forces acting between the microtubule and the MTBD). This is especially important if the E-hooks’ interactions are investigated, since E-hooks can bind (or interact) with electrostatic patches that are far away from the binding interface [28]. Thus, the electrostatic field between the axonemal and cytoplasmic MTBDs, and the microtubule, are shown in Figure 2 (the left panels show the electrostatic filed lines generated by entire tubulins, while the right panels show the electrostatic field lines generated by E-hooks only). Considering the effect of an entire microtubule segment (Figure 2a,c) one can observe that electrostatic field lines make a distinctive arc between β-tubulin chain B and cytoplasmic MTBD, while in the case of an axonemal MTBD, the electrostatic field lines of β-tubulin chain B are not directed toward the MTBD (for chain labeling, see the Methods section). A similar observation can be made comparing Figure 2a,c, where one can clearly see the strong interaction between the β-tubulin chain B E-hook and cytoplasmic MTBD, which is absent in the case of an axonemal MTBD. The situation is different for the β-tubulin of chain D. In both cases, cytoplasmic and axonemal MTBDs, there are strong interactions between β-tubulin chain D and the corresponding MTBD. The same is valid for interactions with E-hooks only (Figure 2b,d). However, the difference is that for axonemal MTBD, these interactions are mostly with the flap region, not with the main body of the MTBD. Another important difference is that for the cytoplasmic MTBD, the interactions (at distance 35 Å) are mostly between microtubules and the binding interface of the MTBD, while for axonemal MTBD, electrostatic interactions involve surface patches away from the binding interface. This is due to the abovementioned charge distribution differences between cytoplasmic and axonemal MTBDs. The axonemal MTBD has many more positively charged patches away from the binding interface than the cytoplasmic one, and they attract negatively charged E-hooks that are capable of reaching further than the binding interface. 

### 2.2. Comparing the Number of Contacts of MTBD-E-Hooks for Axonemal and Cytoplasmic MTBDs at Various Distances

**Figure 3:**
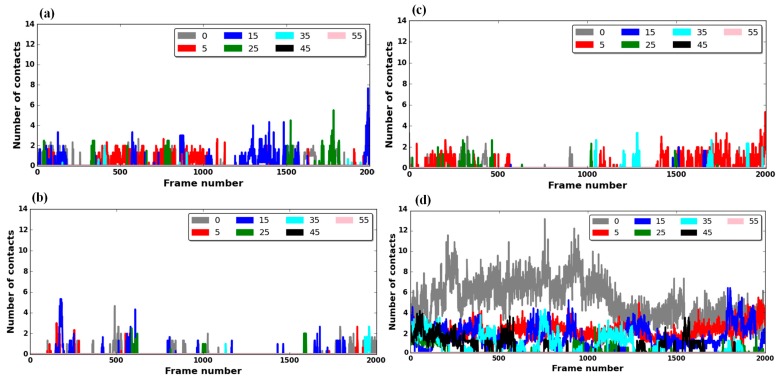
The average number (averaged over three independent molecular dynamics (MD) runs) of E-hook–MTBD contacts as a function of MTBD–microtubule distance and simulation time. (Panels (**a**,**c**)): The average number of E-hook **B**–MTBD contacts at various distances for cytoplasmic and axonemal MTBDs, respectively. (Panels (**b**,**d**)): The average number of E-hook **D**–MTBD contacts at various distances for cytoplasmic and axonemal MTBDs, respectively. For all panels, the bound position (gray plot) corresponds to distance 0 Å, and the MTBD–microtubule distances of 5 Å, 15 Å, 25 Å, 45 Å, and 55 Å are plotted as indicated in the legend. Note that only the snapshots of the last 10 ns (Nano-second) of a total 20 ns of simulation time are plotted.

**Figure 4:**
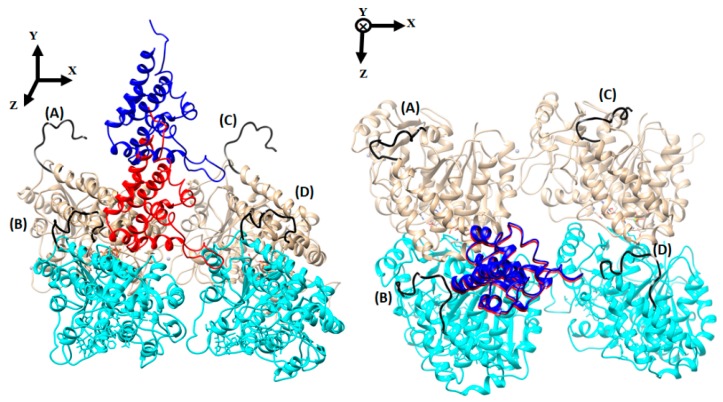
The axonemal MTBD–microtubule segment structure. The side view (left) and top view (right) of two tubulin dimers and an MTBD in the crystallographic position (red) and at a distance of 35 Å (blue). In our structure, we refer to the E-hooks as chains (**A**)–(**D**)**,** where (**B**,**D**) are β-tubulin (cyan) E-hooks, and (**A**,**C**) are the corresponding α-tubulin (brown) E-hooks. All four E-hooks presented in the structure are labeled according to the chain letter of the corresponding tubulin.

To investigate how differently E-hooks interact with axonemal and cytoplasmic MTBDs, we analyzed the number of contacts made between the corresponding E-hooks and the MTBDs (Figure 3). As mentioned above, the MTBD–microtubule distance was varied by offsetting the corresponding MTBD perpendicularly from its bound position by 5 Å, 15 Å, 25 Å, 35 Å, 45 Å, and 55 Å (see the Methods section). We recorded the contact between the E-hooks of four tubulins (chains A, B, C, and D: see the Methods section, Figure 4) and MTBDs, and it was found that only the β-tubulin E-hooks (labeled as B and D, Figure 4) made contact with the corresponding MTBDs. Therefore, below, we present results for the E-hooks associated with β-tubulins only. 

Our results show that there are transient interactions between E-hooks and the MTBD, since the number of contacts between these two partners fluctuates over the simulation time. Moreover, we found that the number of contacts between the cytoplasmic MTBD and E-hook B is much larger than the number with E-hook D; however, the axonemal MTBD makes more contacts with E-hook D instead. This is due to the short distance of the flap region to β-tubulin D (Figure 4). We observed that the number of contacts between the β-tubulin E-hooks and axonemal MTBD are much more than those for the cytoplasmic MTBD (Figure 3). Perhaps, this is because the positively charged flap region of the axonemal MTBD is very close to the β-tubulin surface, and specifically close to the E-hook D. Further, we observed that the E-hooks’ contacts with MTBDs varied as a function of distance without an obvious trend; however, as the distance of the MTBD to the microtubule increased, the number of contacts between the E-hooks and the MTBD decreased. For cytoplasmic and axonemal MTBDs, there are no contacts found at distances larger than or equal to 45 Å and 55 Å, respectively (Figure 3). 

**Figure 5:**
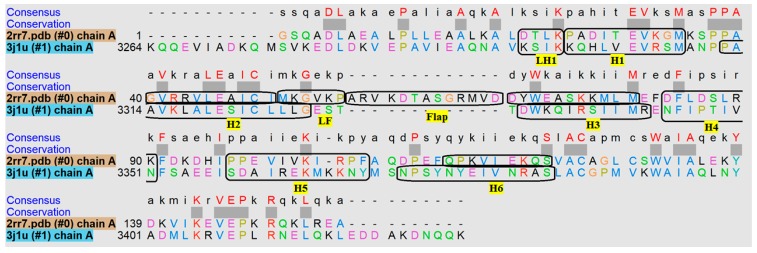
Sequence alignment of axonemal (2rr7.pdb) and cytoplasmic (3j1u.pdb) MTBDs. The six helices are labeled and highlighted in yellow. LH1 and LF stand for the neighboring loop next to H1 and the flap region (The dotted lines and thick grey squares indicate the gap and conserved region between these two proteins amino acid sequence).

To investigate the structural origin of the differences observed above, we superimposed the three-dimensional (3D) structures of axonemal and cytoplasmic MTBDs. Overall, axonemal and cytoplasmic MTBDs consist of six helices, H1–H6, connected to CC1, CC2, which superimpose well (overall root-mean-square deviation (RMSD) = 1.3 Å) [7] (Figure S2), and the main difference is the flap region of the axonemal MTBD. We tabulated the percentage of contacts between E-hooks and each helix of cytoplasmic and axonemal MTBDs (Table 1). First, we found that for the cytoplasmic MTBD, the largest number of contacts was made by residues located in helix H1, the loop connecting H1 to CC1 (LH1), and helix H5. However, for the axonemal MTBD, the largest number of contacts was found to be on the residues of the flexible flap region and the loop next to it (LF), while the helices on the interface almost have not contributed to the interactions with E-hooks. To better understand these differences, the sequence alignment of these two MTBDs is presented in Figure 5. The LH1 in cytoplasmic MTBD has +2 charges (two Lys); however, the same region in the axonemal MTBD is neutral. This explains the preference of negatively charged E-hooks to interact with LH1 in the cytoplasmic MTBD. Furthermore, the larger number of positively charged residues in H1 and H5 of the cytoplasmic MTBD (net charge H1: +1e and H5: +2e) than in the axonemal MTBD (net charge H1: −1e and H5: 0e) is the reason why these helices made contacts with E-hooks in the cytoplasmic MTBD, but not in the axonemal MTBD. In contrast, H2 of the axonemal MTBD makes more contacts than that of the cytoplasmic MTBD, because it carries more positive charges in the axonemal MTBD (net charge H2: +1e in axonemal and H2: 0e in cytoplasmic). At the same time, there are other helices such as H3, H4, and H6 that had no contacts with E-hooks in both MTBDs. While H4 in both MTBDs is a buried helix, and thus it is not accessible, the reason that H3 and H6 do not have a significant number of contacts with E-hooks is most probably due to topology. The most distinguishable difference between cytoplasmic and axonemal MTBDs is the flap region of the axonemal MTBD, which is absent in the cytoplasmic MTBD. 

**Figure 6:**
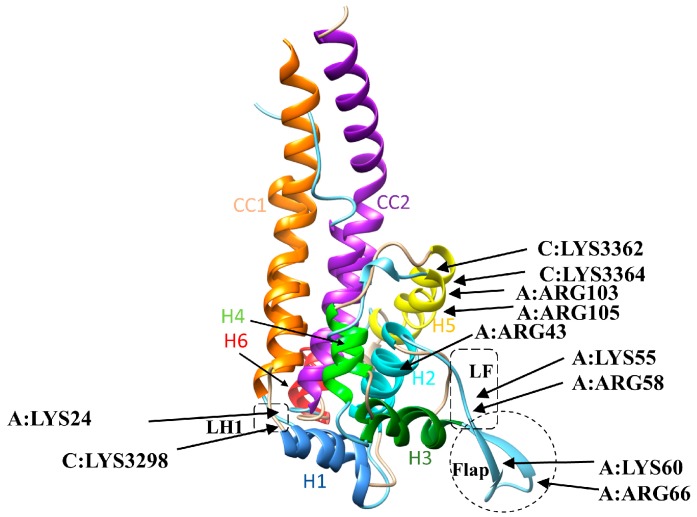
Superimposition of axonemal and cytoplasmic MTBDs along with the residues contributing largely in the contacts with E-hooks. C and A stand for cytoplasmic and axonemal, respectively. (The dotted circle and square indicate the flap region and loop next to the flap region (LF) in axonemal MTBD, respectively).

To identify which residues of MTBDs made contacts with the E-hooks at different distances, we tabulated the total number of contacts (taken from three independent molecular dynamics (MD) runs) for each MTBD residue at different distances (Table 2). In case of the cytoplasmic dynein, we found that the residues LYS3298 (close to H1), LYS3364 (H5), and LYS3367 (H5) make maximum contacts, almost at any MTBD’s distance, with E-hooks B and D, respectively (Figure 6). Residue LYS3298 makes largest number of contacts compared to other residues (Table 2a,b). It should be mentioned that there are some non-charged residues such as SER3296, ILE3297, GLN3300 and ILE3361 that made contacts at particular distances. This is because they are neighbors of positively charged residues interacting with E-hooks via long-range electrostatic interactions. Moreover, a positively charged residue, the LYS3299, was found to interact with E-hook B only at particular distances such as 15 Å and 25 Å. These observations show that the interplay between long-range electrostatic interactions, geometrical and structural constrains, and energetically accessible conformational changes all together contribute to the complex nature of MTBDs—E-hooks binding.

For the axonemal MTBD, we found that the flap region makes most of the contacts with the E-hooks. The residues ARG43, LYS55, ARG58, LYS60, ARG66, and LYS103 are the residues of the axonemal MTBD that made the maximum contacts with E-hook D at almost all distances, along with residue LYS24 (close to H1), which made large contacts with E-hook B at all distances (Figure 6). The rest of the residues had less of a contribution to the interactions with E-hooks; residues such as ARG42, LYS52, ASP61, and ARG105 made few contacts at the bound state only or at specific distances. Also, there are some non-charged residues such as MET51, GLY53, VAL54, PRO56, ALA57, VAL59, THR62, ALA63, SER64, MET67, VAL68, PRO106, and PHE107 that made few contacts with E-hook D at some distances, the reason being the same as that outlined above. 

### 2.3. Conformational Changes of Axonemal and Cytoplasmic MTBDs

**Figure 7:**
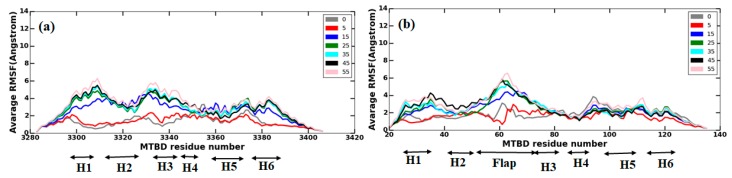
Root-mean-square fluctuation (RMSF) of MTBDs’ residues when MTBDs are located in different distances. Panels (**a**,**b**) show the average RMSF of three independent runs for each residue of cytoplasmic and axonemal MTBDs, respectively.

To investigate how the interactions between E-hooks and the MTBD cause structural changes in the MTBD, the Cα root-mean-square fluctuation (RMSF) values for each residue of the MTBDs (axonemal and cytoplasmic) were calculated (see the Methods section) and averaged over three independent MD runs at various MTBD–microtubule distances: 0 Å, 5 Å, 15 Å, 25 Å, 35 Å, 45 Å, and 55 Å (Figure 7). 

In terms of RMSF, which indicates the magnitude of conformational fluctuations, there is no significant difference between axonemal and cytoplasmic MTBDs (Figure 7). For both MTBDs, the magnitude of conformational fluctuations increases as the distance from the microtubule increases (Figure 7). It indicates that the interactions between the microtubule and the MTBD stabilize the MTBD’s structure and make the MTBDs more rigid upon binding. Furthermore, we labeled H1–H6 and the flap region in Figure 7, and as it is seen, the flap region of the axonemal MTBD was found to be the most flexible structural region at any MTBD distance. For the cytoplasmic MTBD, some helices underwent larger conformational changes than others at some distances; for example, for an MTBD in a bound state (0 A°), the region between H5–H6 showed the largest changes. However, as the MTBD distance increases, other helices close to H1 and H3 also exhibit large conformational changes. 

**Figure 8:**
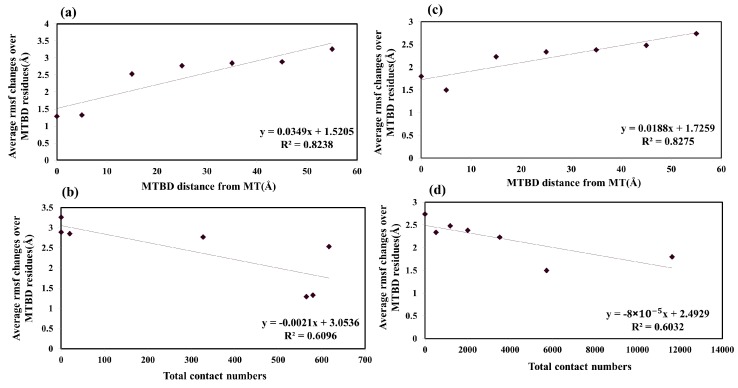
The RMSF changes vs. MTBD distance are shown in panel (**a**,**c**) for cytoplasmic and axonemal MTBDs, respectively. The RMSF changes vs. total contact numbers for the MTBD in each distance are shown in panel (**b**,**d**) for cytoplasmic and axonemal MTBDs, respectively.

To probe whether the conformational fluctuations of MTBDs are correlated with the MTBDs’ distances and their relationship to the number of contacts between the MTBDs and E-hooks, we plotted the average RMSF changes of MTBDs against the MTBDs’ distances and contact numbers (Figure 8). The RMSF is indicative of the structural fluctuations: a large RMSF indicates large structural fluctuations. Very good correlations were obtained (Figure 8). The observations outlined in the paragraph above (see Figure 7) were confirmed. Indeed, as the distance between the corresponding MTBD and microtubule increases (Figure 8a,c) the RMSF increases as well, indicating that MTBDs are more flexible when they are further away from the microtubule. Similarly, as the number of contacts decreases (Figure 8b,d), the MTBDs are more flexible as well (as shown above, the number of contacts decreases as a function of distance, and thus the larger the distance, the smaller the contact number). Thus, the interaction between the MTBD and microtubule, including E-hooks, is a stabilizing factor that rigidifies the MTBD’s structure. The overall observation is that the structural flexibility of the axonemal MTBD increases less as a function of distance than that of cytoplasmic MTBD (see Figure 8, slope of the fitting line). Perhaps this is because the axonemal MTBD senses the microtubule electrostatic field at longer distances than the cytoplasmic, so even at distances between 45–55 Å, it is not completely free (unbound). 

### 2.4. MTBDs Cluster Analysis

Conformational changes of binding partners are common phenomena for protein–protein interactions. To investigate how the interactions between microtubules and MTBDs affect the population of conformational states of isolated MTBDs and bound MTBDs, we carried out a clustering procedure (see the Methods section). The goal was to identify prominent conformational states, assess their population, and compare their population in bound and free states. To have a consistent comparison between the conformational states of bound and unbound cases, we considered the first five most populated clusters in each MTBD–tubulins complex, and elected a representative for each cluster. We tabulated the first five most populated clusters for MTBDs at different distances along with their populations, and at the same time, we compared them with the first five most populated clusters of MTBDs in a free state (Table S1 and Table 2). The RMSD between the representative structure of MTBDs in a bound state and at a particular distance and a free state (without tubulins) was calculated. A small RMSD is used to infer structural similarity. Thus, if a representative structure at a particular MTBD–microtubule distance has a small RMSD with a representative structure of a cluster in a free state, these clusters are considered similar. In contrast, a large RMSD value between representatives in bound and free cases indicates that the conformational states in corresponding clusters in a bound state are quite different from those in a free state. 

It can be observed from the first five clusters of MTBD structures for cytoplasmic and axonemal that the first five clusters of the axonemal MTBD are considerably populated; however, for the cytoplasmic MTBD, the first cluster is predominant, and the rest of the clusters are less populated. Moreover, the RMSD values between the cytoplasmic MTBD in a free state and bound state are less than those of the axonemal MTBD (Table S1 and Table 2). 

### 2.5. E-Hooks Cluster Analysis

Similarly, as done above for MTBDs, we carried out a clustering analysis for the E-hooks involved in contacts with MTBDs. As we observed in Figure 3, the E-hook of chain D for the axonemal MTBD made a large number of contacts; therefore, we analyzed the ensemble structures of the E-hook of chain D for both axonemal and cytoplasmic MTBDs. The results are shown in Table 3, restricting the analysis to a bound state and 25 Å, in order to reduce the amount of data. 

In both MTBDs, the highly populated clusters of E-hooks in a bound state and at a particular distance state tend to have a similarity to the structures of E-hooks in a free state that are sparsely populated. However, the RMSD values between the representative structures are significant; thus, the above comments should be taken with caution. Moreover, for the cytoplasmic MTBD at distance 0 Å, the RMSD values between E-hook structures in bound and free states are smaller than those for the MTBD located at 25 Å. In contrast, for the axonemal MTBD at 25 Å, the minimum RMSD values between E-hooks in bound and free states are smaller than those for at MTBD located at 0 Å. 

### 2.6. Binding Free Energy

The binding energies were obtained using all snapshots in which E-hooks and MTBDs made contacts (Table 4). It was found that the binding energy for the cytoplasmic MTBD is more favorable than that of the axonemal MTBD in both the presence of E-hooks and without E-hooks. This finding can be attributed to the difference in the functionalities of cytoplasmic and axonemal dyneins. Indeed, axonemal dynein is responsible for beating cilia, and to do that, it needs to have a weak binding to the microtubule in order to be able to disassociate quickly. 

## 3. Materials and Methods

In our study, two cases will be investigated: (a) a microtubule-binding domain (MTBD) of cytoplasmic dynein in a low affinity state, bound to the microtubule segment, and (b) an axonemal MTBD bound to the same microtubule segment. The 3D models were created as described below (Figure 4 and Figure S1). The reason for selecting for this study the low affinity state of cytoplasmic dynein is the structural similarity between the cytoplasmic MTBD in this state and the axonemal MTBD. Indeed, the Cα root-mean-square deviation (RMSD) between the key interfacial helices (H1, H3, and H6—see Figure S1a,b) of cytoplasmic in a low affinity state and axonemal MTBD is 3.16 Å, while it is 6.17 Å for a high affinity state of cytoplasmic MTBD. 

The crystal structure of the cytoplasmic MTBD in a low affinity state (Mus musculus), bound to alpha-beta tubulin from Bos taurus, is available in the Protein Data Bank [34] (PDB ID 3J1U [12]). Since the resolution of alpha beta tubulin in the mentioned structure is low (9.7Å), we replaced it with the refined tubulin structure from the same organism (PDB ID 1JFF [35]) with a higher resolution of 3.5 Å. The microtubule is a long filament composed of alpha-beta tubulins; therefore, to account for the effect of the neighboring tubulins, we modeled a microtubule segment of two dimers using a rotation and translation matrix of a protein structure (PDB ID 3J2U [36]) (Figure S3). (Note that various structural segments were tried that were composed of more than two dimers, but it was found that no E-hooks–MTBD contacts were made in the MD simulations. Since one of the major goals of this study is to investigate MTBD–E-hooks interactions, these structural segments were not analyzed. Details are provided in [28].

Due to the lack of a crystallographic structure of the axonemal MTBD bound to tubulins, we built a model of axonemal MTBD bound to the microtubule as follows. The NMR (Nuclear Magnetic Resonance) structure of axonemal MTBD (PDB ID 2RR7 [7] from Chlamydomonas reinhardtii) was used, which is structurally similar to cytoplasmic MTBD in a low affinity state (Figure S1). Thus, using structural imposition, the structure of the cytoplasmic MTBD in a low affinity state bound to the α and β tubulins described above was replaced by axonemal MTBD (Figure 4), and the possible clashes between the axonemal MTBD and tubulins were removed.

The α and β tubulins have an intrinsically disordered region located at the C-terminal domain, which is typically called an E-hook [15]. Knowing the amino acid sequence of E-hooks, in the α-tubulin (VGVDSVEGEGEEEGEEY) and β-tubulin (DATADEQGEFEEEGEEDEA) of Bos taurus, we generated their 3D structures using Profix [37]. They are labeled and colored black in Figure 4 and Figure S1. 

To model the association of the corresponding MTBD, axonemal and cytoplasmic, with the microtubule, including E-hooks, the MTBD was offset at different distances away from the microtubule. To do so, the structure of the corresponding MTBDs bound to the microtubule segment was moved away from the bound position along the axis perpendicular to the microtubule by 5 Å, 15 Å, 25 Å, 35 Å, 45 Å, and 55 Å, respectively (Figure 4 and Figure S1 show MTBDs at bound position and 35 Å away). 

### 3.1. Molecular Dynamics (MD) Simulations

The energy minimization and molecular dynamics (MD) simulations were done with NAMD (Nanoscale Molecular Dynamics) [38]. The temperature and the ion concentration were assigned at 300 K and 0.15 M, respectively. The generalized Born (GB) [39] implicit solvent model and the CHARMM (Chemistry at Harvard Macromolecular Mechanics) force field [40] were used. It should be mentioned that GB is an approximation of explicit water simulations, and may miss some of the explicit water effects. The cutoff for Born radii was set at 12.0 Å. MD simulations were done three times with different atomic velocities for a total of 20 ns, and all analyses were done for the last 10 ns of MD runs by VMD (Visual Molecular Dynamics) [41]. 

The structural segment of the microtubule and the MTBD are part of large molecular complex: the entire microtubule and entire dynein molecule. To reduce the effect of missing neighboring tubulins, in the MD simulations, we applied harmonic restraints on all the residues of the tubulins representing microtubule segments (Figure 4 and Figure S1) except for the E-hooks. Moreover, MTBDs (cytoplasmic PDB ID 3J1U [12] and axonemal PDB ID 2RR7 [7]) are huge protein (dynein) domains, and were obtained with a part of the coiled-coil stalk domain (CC1 and CC2) at a particular conformation. To keep the CC1/CC2 conformation, we restrained several residues at the top of these helices in both cytoplasmic and axonemal MTBDs [42] (residues 1 to 3 in CC1 and 142 to 155 in CC2 for axonemal MTBD; and residues 3264 to 3266 in CC1 and 3425 to 3427 in CC2 for cytoplasmic MTBD). To do MD simulation for MTBDs in an isolated state, we used the same parameters and constraints for MTBDs without microtubules for a total of 20 ns in three independent MD runs. 

### 3.2. Electrostatic Potential, Electrostatic Field Lines, and Binding Energy

To calculate the 3D distribution of electrostatic potential, we used DelPhi, which numerically solves the Poisson–Boltzmann equation (PBE) [43]. The parameters used for the calculations were CHARMM charges and radii; resolution: 2 grids/Å (Angstrom); the perfil of 70; and the dielectric constants of 2 and 80 for protein and water, respectively. The salt concentration was assigned at an ionic strength of I = 0.15 M. For obtaining electrostatic potential, we solved the linear PBE. Information regarding the parameters is available in the DelPhi manual (http://compbio.clemson.edu/downloadDir/delphi/delphi_manual.pdf). The electrostatic field lines were generated from the calculated electrostatic potential and visualized by VMD [41].

The molecular mechanics/generalized Born (MM/GB) method was applied to calculate the binding free energies (ΔE_binding_) of the corresponding MTBD to the segment of the microtubule for all the snapshots of MD simulation in which E-hooks and MTBDs made contact. Moreover, for the corresponding snapshots, the binding energies of MTBDs and the segment of the microtubule without E-hooks were calculated using rigid-body protocol such that the structures of the MTBD and the microtubule segment were kept rigid, as they were in the bound state. Thus:

ΔE_binding =_ E_Complex_ − E_MTBD_ − E_MT_ (1)

E_complex,_ E_MTBD,_ and E_MT_ (MT stands for microtubule) are the molecular mechanic (MM/GB) energy of the complex, the MTBD, and the microtubule, respectively. Where:

E_MM/GB =_ E_elec_ + E_VDW_ (2)

E_elec_ and E_VDW_ are the total electrostatic (Coulomb and generalized Born energies) and van der Waals energies. 

### 3.3. Analysis of Contacts

We counted the number of contacts between MTBDs and E-hooks to investigate the interactions between MTBDs and E-hooks. To do so, any event at which a heavy atom of E-hooks is within a 4-Å distance from an atom of MTBD was considered as a contact. We obtained the number of contacts and residues contributing to contact using VMD [41]. In the Results section, the contact number is averaged over three independent MD trajectories. 

### 3.4. Analysis of Conformational States

We analyzed the Cα root-mean-square deviation (RMSD) and Cα root-mean-square fluctuation (RMSF) of the MTBDs’ residues and residues within E-hooks using VMD [41]. It should be pointed out that the average Cα RMSD value per residue indicates the *conformational change* of this particular residue with respect to the initial structure (built model). In contrast, the Cα RMSF indicates the *conformational fluctuations* of a residue with respect with the averaged structure over all the frames. Moreover, we calculated the average of the RMSF values of all the residues within either MTBDs or E-hooks as:

(3) $$\mathrm{RMSF}=\frac{\sum_{i=residue}{\left(\mathrm{RMSF}\right)}_{i}}{total\;number\;of\;residues}$$

To investigate how the population rate of conformational states of MTBDs (axonemal and cytoplasmic) and E-hooks in unbound states change upon binding to the microtubule, we clustered all of the corresponding snapshots obtained via three different runs. This was done for MTBDs in bound and unbound states, and for MTBDs located at different distances: 0 Å, 5 Å, 15 Å, 25 Å, 35 Å, 45 Å, and 55 Å. Cluster analysis was done using the “cluster” tool in Gromacs [44,45] by the Daura algorithm [46] applying a C alpha root-mean-square deviation (RMSD) cutoff of 2 Å. In our study, we considered only the first five most populated clusters. This tool allows us to collect a representative for each cluster that is the most common structure in each pool. The same procedure was used for analyzing the conformational states of E-hooks, except we used a smaller cutoff, 1.5 Å, since E-hooks have much less residues compared with MTBDs, which allowed having multiple populated clusters for E-hooks (see Ref. [28]).

## 4. Conclusions

The investigation focused on revealing the role of electrostatics in the association of cytoplasmic and axonemal MTBD with microtubules, with the goal of attributing the differences to the functionality (processivity versus beating) of these MTBDs. It is anticipated that processivity will require that the corresponding domain is tightly bound to the microtubule, and thus stays associated with it for a longer time (as we demonstrated for cytoplasmic MTBD, the binding is correlated with processivity [42]). In contrast, the beating should require a weak association, but the interactions should be exerted for a longer time. 

While the amino acid sequence of axonemal and cytoplasmic MTBDs and their 3D structures (excluding the flap region) are quite similar, their charge distributions are different. In cytoplasmic MTBD, the positively charged residues are clustered mostly along the binding interface with the microtubule, while in the axonemal MTBD, they are scattered over the entire MTBD. This results in distinctively different electrostatic interactions between the microtubule and the corresponding MTBD. Our work indicates that the electrostatic interactions for axonemal MTBD are longer-ranged compared with cytoplasmic MTBD (the axonemal MTBD makes contacts with E-hook even at the distance of 45 Å, while the cytoplasmic MTBD does not). 

The analysis of the conformational changes of MTBDs as a function of distance from the microtubule indicated that structural fluctuations increase as the distance from the microtubule increases. This was found for both cytoplasmic and axonemal MTBDs. However, the conformational flexibility of the axonemal MTBD increases less as a function of distance compared with cytoplasmic MTBD. This was attributed to the above-mentioned long-range electrostatic interactions, so the axonemal MTBD is not completely free (unbound), even at distances between 45–55 Å. 

Our previous work [28] on cytoplasmic MTBD (in a high affinity state) showed that the binding induces a change of the population of pre-existing clusters of E-hooks. Thus, we showed that there is a tendency for the most occupied cluster in a bound state to be similar to the least occupied cluster in a free state and vice versa. A similar effect was observed in this study; however, the tendency is much weaker. 

We do not see such an effect for both cytoplasmic and axonemal MTBDs. The clusters in a bound state are very different from the clusters in a free state. It seems to us that the binding rigidifies both MTBDs, and they adopt a conformation that is not seen in a free state. Indeed, we showed that the conformational dynamics of MTBDs decrease as the distance to the microtubule decreases, or as the number of contacts increases.

## Figures and Tables

**Table 1:** The absolute number of contacts and their percentage for each helix of cytoplasmic and axonemal MTBDs at 0 Å, respectively (LF stands for “loop next to flap region”, and LH1 stands for “loop next to H1”).

**Cytoplasmic MTBD**	**Number of Contacts**	**Percentage of Contacts**
LH1	401	70.97%
H1	5	0.88%
H2	0	0.00%
H3	0	0.00%
H4	0	0.00%
H5	159	28.14%
H6	0	0.00%
**Axonemal MTBD**	**Number of Contacts**	**Percentage of Contacts**
LH1	95	0.82%
H1	0	0.00%
H2	1871	16.10%
H3	0	0.00%
H4	0	0.00%
H5	1081	9.30%
H6	0	0.00%
Flap	4780	41.11%
LF	3799	32.67%

**Table 2:** The total number of contacts between the corresponding E-hook and MTBD residues. (**a**,**b**): MTBD residues interacting with E-hook B for cytoplasmic and axonemal MTBD, respectively; (**b**,**d**): The same with E-hook D for cytoplasmic and axonemal MTBD, respectively.

**(a)**						
**Cytoplasmic MTBD residues interacting with E-hook B**	**MTBD in bound state**	**MTBD in 5 A°**	**MTBD in 15 A°**	**MTBD in 25 A°**	**MTBD in 35 A°**	
LYS3295	16	7	1	0	0	
SER3296	16	0	1	0	0	
ILE3297	6	0	0	0	0	
LYS3298	363	505	334	97	4	
LYS3299	4	0	30	162	14	
GLN3300	1	0	58	68	2	
**(b)**						
**Cytoplasmic MTBD residues interacting with E-hook D**	**MTBD in bound state**	**MTBD in 5 A°**	**MTBD in 15 A°**			
ILE3361	4	1	0			
LYS3364	141	39	68			
LYS3367	14	28	125			
**(c)**						
**Axonemal MTBD residues interacting with E-hook B**	**MTBD in bound state**	**MTBD in 5 A°**	**MTBD in 15 A°**	**MTBD in 25 A°**	**MTBD in 35 A°**	
THR22	14	5	0	0	0	
LEU23	8	0	0	0	0	
LYS24	73	517	16	50	5	
THR29	0	0	0	4	4	
**(d)**						
**Axonemal MTBD residues interacting with E-hook D**	**MTBD in bound state**	**MTBD in 5 A°**	**MTBD in 15 A°**	**MTBD in 25 A°**	**MTBD in 35 A°**	**MTBD in 45 A°**
ARG42	51	0	0	0	0	241
ARG43	1820	0	155	0	0	0
MET51	659	0	0	0	0	0
LYS52	209	0	1	0	0	0
GLY53	233	1	13	0	0	0
VAL54	388	0	4	0	0	0
LYS55	1715	7	11	0	26	0
PRO56	595	0	0	0	0	0
ALA57	966	0	0	0	2	0
ARG58	2126	0	1269	256	0	177
VAL59	4	0	1	0	0	0
LYS60	35	0	563	15	628	104
ASP61	1	2	2	1	1	5
THR62	0	0	2	25	1	0
ALA63	1	213	37	37	3	1
SER64	0	457	20	6	147	27
GLY65	0	10	3	3	0	5
ARG66	1629	4492	1352	105	1189	620
MET67	18	1	2	1	0	1
VAL68	0	6	0	1	0	4
LYS103	208	5	56	7	0	0
ARG105	774	0	0	0	0	0
PRO106	24	0	0	0	0	0
PHE107	75	0	3	1	0	0

**Table 3:** Cluster analysis of the conformational states for the E-hook in chain D. First five populated clusters of the E-hook in chain D for an isolated state (microtubule only) and a bound state (MTBD bound to microtubule). The root-mean-square deviation (RMSD) values between each representative of E-hooks in a free state and E-hooks in a bound state were shown in each block in Å, and the minimum values were bolded.

**^3^ (a) Cytoplasmic MTBD–microtubule distance = 0 Å**					
**Free-E-hook**	**Cluster1 (51.2%)**	**Cluster2 (20.85)**	**Cluster3 (8.6%)**	**Cluster4 (7.95%)**	**Cluster5 (4.45%)**
Cluster1 (59.8%)	5	5.2	5.17	4.79	5.86
Cluster2 (8.1%)	3.83	3.56	3.58	3.33	**4.1**
Cluster3 (6.5%)	3.32	3.27	3.21	4.08	5.39
Cluster4 (5.3%)	2.9	3	3.03	3.5	5.57
Cluster5 (4.7%)	**2.68**	**2.28**	**2.31**	**3.06**	4.3
**^3^ (b) Cytoplasmic MTBD–microtubule distance = 25 Å**					
**Free-E-hook**	**Cluster1 (33%)**	**Cluster2 (19.1%)**	**Cluster3 (12.65%)**	**Cluster4 (9.32%)**	**Cluster5 (5.05%)**
Cluster1 (59.8%)	7.04	4.91	6.31	6.35	5.82
Cluster2 (8.1%)	6.67	3.28	**4.66**	5.68	5.94
Cluster3 (6.5%)	6.35	3.37	5.98	6.29	6.36
Cluster4 (5.3%)	6.37	2.87	5.5	5.94	6.01
Cluster5 (4.7%)	**5.63**	**2.8**	5.29	**5.65**	**5.9**
**^3^ (c) Axonemal MTBD–microtubule distance = 0 Å**					
**Free-E-hook**	**Cluster1 (31.54%)**	**Cluster2 (16.12%)**	**Cluster3 (7.44%)**	**Cluster4 (4.94%)**	**Cluster5 (4.24%)**
Cluster1 (59.8%)	4.35	5.83	5.58	4.51	5.39
Cluster2 (8.1%)	3.25	3.96	**4.62**	4.99	**4.28**
Cluster3 (6.5%)	2.88	4.08	5.69	3.88	5.17
Cluster4 (5.3%)	**2.24**	**3.89**	4.92	**3.14**	4.69
Cluster5 (4.7%)	3.47	4.28	5.53	4.72	4.9
**^3^ (d) Axonemal MTBD–microtubule distance = 25 Å**					
**Free-E-hook**	**Cluster1 (36.15%)**	**Cluster2 (27.3%)**	**Cluster3 (16.35%)**	**Cluster4 (6.65%)**	**Cluster5 (4.8%)**
Cluster1 (59.8%)	5.01	4.98	5.18	4.87	4.73
Cluster2 (8.1%)	3.6	3.84	4.25	3.5	3.57
Cluster3 (6.5%)	2.21	3.42	2.95	2.72	2.67
Cluster4 (5.3%)	**2.2**	**2.87**	**2.66**	**2.37**	**2.15**
Cluster5 (4.7%)	3.18	2.97	3.62	2.86	3.35

**Table 4:** Binding energies (∆E_binding_) and the corresponding components for cytoplasmic and axonemal MTBDs–microtubule contact in the presence of E-hooks and without E-hooks. All energies are calculated as a sum of ΔE_elec_ (elec stands for electrostatics) and ΔE_VDW_ (VDW stands for van der Waals) (see Methods for details). Energies are provided in kcal/mol.

Binding Free Energy	Complex	MTBD	Free-Tubulins	Δ_binding_ ± Standard Deviation
Cytoplasmic MTBD–microtubule with E-hooks	−22,243.6	−3906.06	−18,296.7	−40.84 ± 7.56
Cytoplasmic MTBD–microtubule without E-hooks	−20,113.2	−3906.06	−16,227.9	20.76 ± 6.71
Axonemal MTBD–microtubule with E-hooks	−21,851.2	−3520.25	−18,302.2	−28.75 ± 6.33
Axonemal MTBD–microtubule without E-hooks	−19,726	−3520.25	−16,230.8	25.05 ± 10.29

## Supplementary Materials

Supplementary materials can be found at https://www.mdpi.com/1422-0067/20/5/1090/s1.

## Abbreviations

VDW	Van der Waals
Å	Angstrom
RMSF	Root-Mean-Square Fluctuation
PBE	Poisson-Boltzmann equation
MM/GB	Molecular Mechanics Generalized Born
MT	Microtubule
RMSD	Root-Mean-Square Deviation
PDB	Protein Data Bank
MD	Molecular Dynamics
E-hook	Intrinsic disordered region in C-terminal domain of tubulins
MTBD	Microtubule Binding Domain
ATP	Adenosine Triphosphate
KT/e	KT/e is electrostatic potential unit.
Ns	Nano second
Elect	Electrostatics
VMD	Visual Molecular Dynamics
NMR	Nuclear Magnetic Resonance
NAMD	Nanoscale Molecular dynamics
CHARMM	Chemistry at Harvard Macromolecular Mechanics
