# Supplementary material for: Processivity vs. Beating: Comparing Cytoplasmic and Axonemal Dynein Microtubule Binding Domain Association with Microtubule
Source: Int J Mol Sci. 2019 Mar 3;20(5):1090. doi: 10.3390/ijms20051090 (PMC6429364; doi:10.3390/ijms20051090)
Supplement: Supplementary file 1 [file ijms-20-01090-s001.pdf]

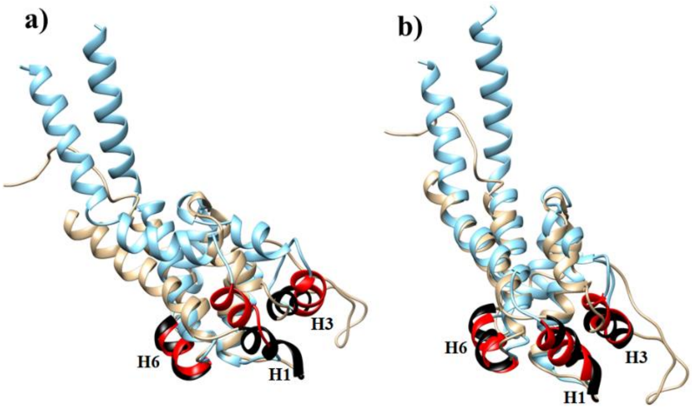

Figure S1. (**a**) The superimposition of cytoplasmic in high affinity state and axonemal MTBD and (**b**) the one for cytoplasmic in low affinity state and axonemal MTBD. The H1, H3 and H6 which are in the interface labeled and colored in black and red. The cytoplasmic MTBDs in both figures are blue and axonemal MTBD are cyan.

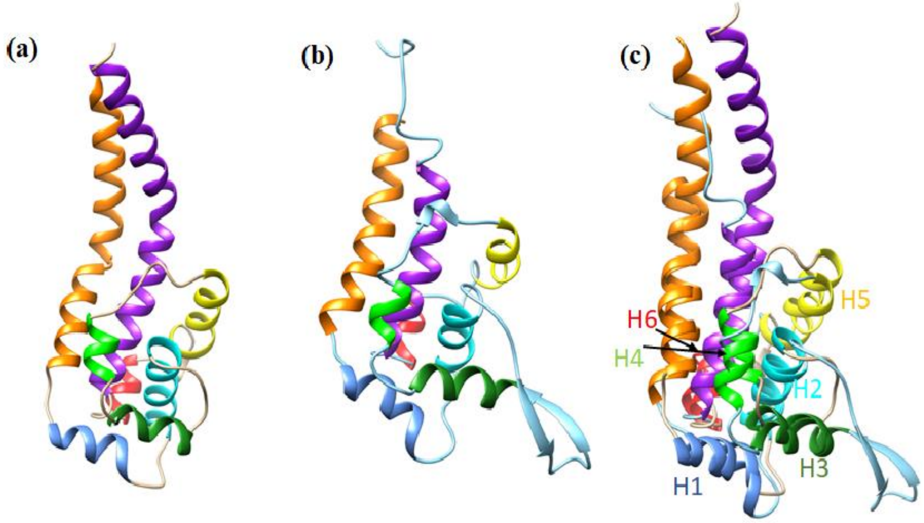

Figure S2. MTBDs secondary structure elements (SSE), including CC1 and CC2. Panel (**a**) represents cytoplasmic (in weak binding state) and panel (**b**) axonemal MTBDs. Panel(**c**) superimposition of cytoplasmic and axonemal MTBDs with colored helices.

Table S1. Clustering analysis of conformational states of cytoplasmic MTBD for different distances of MTBD from tubulins, 0, 5, 15, 25, 35, 45, and 55 Å. The population percentage of each cluster is shown in the parenthesis. First five most populated clusters for MTBD in free state were shown in the first column and those for MTBD in bound state and at a particular distance were shown in the first row. The RMSD values (in terms of Å) between a reprehensive of each cluster in isolated state with one of bound state were provided as well and the minimum RMSD value for each cluster in bound state is bolded.

**Bounded MTBD at 0Å ▶**<br>**Free state MTBD▼**	**Cluster1 (44.4%)**	**Cluster2 (21.5%)**	**Cluster3 (16%)**	**Cluster4 (10.3%)**	**Cluster5 (5.1%)**
Cluster1(71.1%)	**4.148**	**3.721**	**4.408**	**4.501**	**4.099**
Cluster2(17.5%)	4.553	4.191	4.792	4.966	4.465
Cluster3(8.1%)	4.696	4.055	4.855	4.857	4.715
Cluster4(3%)	4.23	4.105	4.477	4.779	4.122
Cluster5(2%)	5.014	4.437	5.234	5.275	4.977
**Bounded MTBD at 5Å ▶**<br>**Free state MTBD▼**	Cluster1 (38.6%)	Cluster2 (25%)	Cluster3 (19.5%)	Cluster4 (4.7%)	Cluster5 (3.8%)
Cluster1(71.1%)	**2.94**	**2.921**	**3.786**	4.231	**3.596**
Cluster2(17.5%)	3.47	3.235	4.07	4.349	3.946

Cluster3(8.1%)	3.374	3.29	4.321	4.67	4
Cluster4(3%)	3.43	3.5	3.88	**4.17**	4.04
Cluster5(2%)	3.803	3.729	4.44	4.775	4.402
**Bounded MTBD at 15Å** ▶ **Free state MTBD▼**	Cluster1 (34.5%)	Cluster2 (27.1%)	Cluster3 (14.6%)	Cluster4 (11.4%)	Cluster5 (3%)
Cluster1(71.1%)	**2.037**	**2.577**	3.797	**2.098**	**2.937**
Cluster2(17.5%)	2.336	2.858	4.01	2.495	3.195
Cluster3(8.1%)	2.214	2.666	4.259	2.442	3.086
Cluster4(3%)	2.69	3	**3.71**	2.71	3.11
Cluster5(2%)	2.76	3.46	4.56	2.95	3.73
**Bounded MTBD at 25Å** ▶ **Free state MTBD▼**	Cluster1 (33.4%)	Cluster2 (32.7%)	Cluster3 (6.3%)	Cluster4 (6%)	Cluster5 (4.4%)
Cluster1(71.1%)	**2.41**	**2.32**	**2.459**	2.251	**2.83**
Cluster2(17.5%)	2.68	2.381	2.727	**2.077**	3.398
Cluster3(8.1%)	2.803	2.544	2.581	2.648	2.934
Cluster4(3%)	2.935	2.848	3.113	2.892	3.165
Cluster5(2%)	3.025	2.866	2.92	2.14	3.85
**Bounded MTBD at 35Å** ▶ **Free state MTBD▼**	Cluster1 (33.2%)	Cluster2 (22%)	Cluster3 (14.6%)	Cluster4 (10.8%)	Cluster5 (5.5%)
Cluster1(71.1%)	**2**	**2.738**	2.91	2.63	**2.237**
Cluster2(17.5%)	2.28	3.034	2.72	2.59	2.54
Cluster3(8.1%)	2.503	2.843	**2.59**	**1.829**	2.91
Cluster4(3%)	3.235	3.201	3.178	2.76	2.82
Cluster5(2%)	2.625	2.941	3.047	3.18	2.96
**Bounded MTBD at 45Å** ▶ **Free state MTBD▼**	Cluster1 (29.5%)	Cluster2 (21.3%)	Cluster3 (12.4%)	Cluster4 (8.6%)	Cluster5 (4.2%)
Cluster1(71.1%)	2.56	**2.038**	**2.759**	**2.608**	2.89
Cluster2(17.5%)	**2.21**	2.634	3.027	3.05	3.56
Cluster3(8.1%)	3.06	2.043	2.881	2.333	**2.85**
Cluster4(3%)	2.77	2.401	3.413	2.621	3.66
Cluster5(2%)	3.12	2.941	3.22	3.18	3.24
**Bounded MTBD at 55Å** ▶ **Free state MTBD▼**	Cluster1 (35.2%)	Cluster2 (11.7%)	Cluster3 (8.6%)	Cluster4 (7.7%)	Cluster5 (5.5%)
Cluster1(71.1%)	**2.046**	3.879	**3.538**	**2.377**	3.23
Cluster2(17.5%)	2.513	4.472	4.134	2.743	3.709
Cluster3(8.1%)	2.08	**3.597**	3.543	2.466	**2.429**
Cluster4(3%)	2.77	4.078	3.901	3.275	3.77
Cluster5(2%)	2.79	4.947	4.441	2.76	4.08

Table S2. Clustering analysis of conformational states of axonemal MTBD for different distances of MTBD from tubulins, 0, 5, 15, 25, 35, 45, and 55 Å. The population percentage of each cluster is shown in the parenthesis. First five most populated clusters for MTBD in free state were shown in the first column and those for MTBD in bound state and at a particular distance were shown in the first row. The RMSD values (in terms of Å) between a reprehensive of each cluster in isolated state with one of bound state were provided as well and the minimum RMSD value for each cluster in bound state is bolded.

**Bounded MTBD at 0Å** ▶ **Free state MTBD▼**	**Cluster1 (35.2%)**	**Cluster2 (16.4%)**	**Cluster3 (11.1%)**	**Cluster4 (8%)**	**Cluster5 (5.1%)**
Cluster1(36.6%)	**4.25**	**5.56**	**5.09**	**5.45**	5.3
Cluster2(27.1%)	4.75	5.82	5.6	5.76	**5.09**
Cluster3(15.6%)	5.16	5.64	5.14	5.36	5.1

Cluster4(9%)	4.52	5.82	5.15	5.25	5.93
Cluster5(3.1%)	4.9	5.75	5.16	5.29	5.4
**Bounded MTBD at 5Å ▶** **Free state MTBD▼**	Cluster1 (59%)	Cluster2 (25%)	Cluster3 (8.2%)	Cluster4 (4.3%)	Cluster5 (2%)
Cluster1(36.6%)	**4.16**	**4.26**	**4.13**	**4.25**	**3.96**
Cluster2(27.1%)	4.29	4.36	4.31	4.34	4.27
Cluster3(15.6%)	4.87	4.54	5.06	4.69	4.84
Cluster4(9%)	4.94	4.75	5.02	4.86	4.95
Cluster5(3.1%)	4.64	4.31	4.76	4.42	4.61
**Bounded MTBD at 15Å ▶** **Free state MTBD▼**	Cluster1 (27%)	Cluster2 (26.5%)	Cluster3 (11%)	Cluster4 (7.2%)	Cluster5 (4%)
Cluster1(36.6%)	5.24	**3.54**	**3.36**	**4.32**	**4.83**
Cluster2(27.1%)	**2.74**	4.54	4.64	4.47	5.19
Cluster3(15.6%)	5	5.49	5.65	5.8	6.43
Cluster4(9%)	4.22	5.07	5.33	4.93	5.93
Cluster5(3.1%)	3.06	5.06	5.15	5.33	5.07
**Bounded MTBD at 25Å ▶** **Free state MTBD▼**	Cluster1 (29%)	Cluster2 (18.7%)	Cluster3 (15.4%)	Cluster4 (12.7%)	Cluster5 (4%)
Cluster1(36.6%)	**3.74**	**4.63**	**3.34**	**4.01**	4.05
Cluster2(27.1%)	4.54	5.67	3.75	5.7	**3.46**
Cluster3(15.6%)	4.71	6.14	3.98	6.22	4.35
Cluster4(9%)	4.75	6.71	3.98	6.69	3.85
Cluster5(3.1%)	4.32	5.77	3.6	5.79	3.98
**Bounded MTBD at 35Å ▶** **Free state MTBD▼**	Cluster1 (37%)	Cluster2 (22%)	Cluster3 (16.5%)	Cluster4 (8.7%)	Cluster5 (6.2%)
Cluster1(36.6%)	5.49	4.63	4.35	4.93	4.86
Cluster2(27.1%)	5.03	4.19	**3.78**	4.67	4.24
Cluster3(15.6%)	**3.9**	3.52	4.23	**3.85**	**3.47**
Cluster4(9%)	4.47	3.61	3.96	4.61	3.77
Cluster5(3.1%)	4	**3.49**	4	4.12	3.48
**Bounded MTBD at 45Å ▶** **Free state MTBD▼**	Cluster1 (30%)	Cluster2 (17.6%)	Cluster3 (13%)	Cluster4 (7.4%)	Cluster5 (5.1%)
Cluster1(36.6%)	**2.67**	4.62	3.95	**3.28**	4
Cluster2(27.1%)	4.26	**3.89**	3.78	4.38	**3.97**
Cluster3(15.6%)	2.9	4.36	3.68	5.3	4.76
Cluster4(9%)	2.97	4.2	3.98	5.07	4.26
Cluster5(3.1%)	4.4	4.02	**3.3**	4.83	4.54
**Bounded MTBD at 55Å ▶** **Free state MTBD▼**	Cluster1 (38%)	Cluster2 (16.7%)	Cluster3 (14.2%)	Cluster4 (8.6%)	Cluster5 (3.2%)
Cluster1(36.6%)	3.79	4.34	4.11	4.36	3.91
Cluster2(27.1%)	3.46	3.73	3.9	4.16	3.53
Cluster3(15.6%)	3.75	3.83	4.14	4.13	3.93
Cluster4(9%)	3.56	4.02	4.16	4.04	3.84
Cluster5(3.1%)	**3.14**	**3.47**	**3.66**	**3.64**	**3.45**

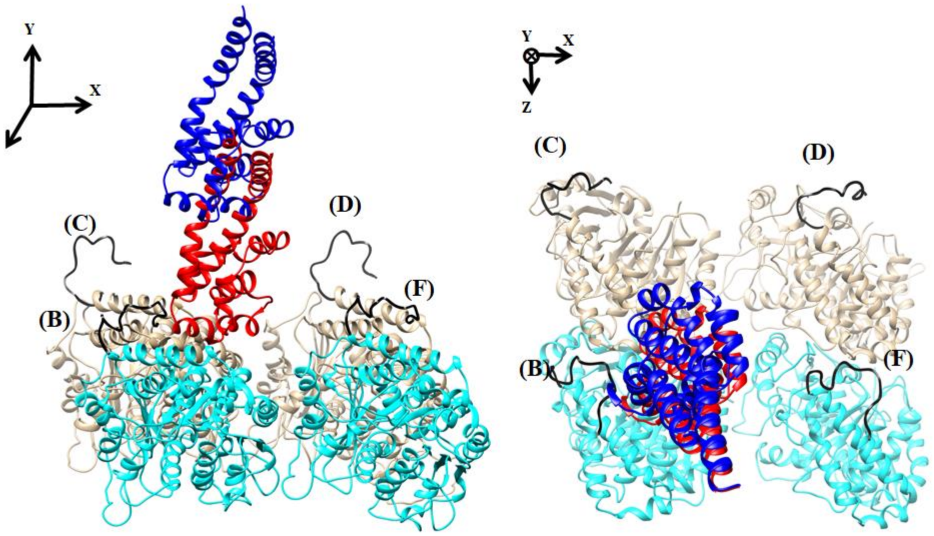

Figure S3. The Cytoplasmic MTBD in β registry - microtubule segment structure**.** The side view (left) and top view (right) of two tubulin dimers and a MTBD in crystallographic position (red) and at a distance of 35 Å (blue). In our structure, we refer to the E-hooks as chains A, B, C, and D where B and D are β-tubulin (cyan) E-hooks, and A and C the corresponding α-tubulin (brown) E-hooks. All four E-hooks presented in the structure are labeled according to the chain letter of the corresponding tubulin.
